# Deferred Pre-Emptive Switch from Calcineurin Inhibitor to Sirolimus Leads to Improvement in GFR and Expansion of T Regulatory Cell Population: A Randomized, Controlled Trial

**DOI:** 10.1371/journal.pone.0075591

**Published:** 2013-10-11

**Authors:** Dinesh Bansal, Ashok K. Yadav, Vinod Kumar, Mukut Minz, Vinay Sakhuja, Vivekanand Jha

**Affiliations:** 1 Department of Nephrology, Postgraduate Institute of Medical Education and Research, Chandigarh, India; 2 Department of Renal Transplant Surgery, Postgraduate Institute of Medical Education and Research, Chandigarh, India; 3 Department of Regenerative and Translational Medicine, Postgraduate Institute of Medical Education and Research, Chandigarh, India; 4 George Institute of International Health, Hyderabad, India; University of Colorado School of Medicine, United States of America

## Abstract

**Background:**

Measures to prevent chronic calcineurin inhibitor (CNI) toxicity have included limiting exposure by switching to sirolimus (SIR). SIR may favorably influence T regulator cell (T_reg_) population. This randomized controlled trial compares the effect of switching from CNI to SIR on glomerular filtration rate (GFR) and T_reg_ frequency.

**Methods:**

In this prospective open label randomized trial, primary living donor kidney transplant recipients on CNI-based immunosuppression were randomized to continue CNI or switched to sirolimus 2 months after surgery; 29 were randomized to receive CNI and 31 to SIR. All patients received mycophenolate mofetil and steroids. The main outcome parameter was estimated GFR (eGFR) at 180 days. T_reg_ population was estimated by flowcytometry.

**Results:**

Baseline characteristics in the two groups were similar. Forty-eight patients completed the trial. At six months, patients in the SIR group had significantly higher eGFR as compared to those in the CNI group (88.94±11.78 vs 80.59±16.51 mL/min, p = 0.038). Patients on SIR had a 12 mL/min gain of eGFR of at the end of six months. Patients in the SIR group showed significant increase in T_reg_ population at 30 days, which persisted till day 180. There was no difference in the adverse events in terms of number of acute rejection episodes, death, infections, proteinuria, lipid profile, blood pressure control and hematological parameters between the two groups. Four patients taking SIR developed enthesitis. No patient left the study or switched treatment because of adverse event.

**Conclusions:**

A deferred pre-emptive switch over from CNI to SIR safely improves renal function and T_reg_ population at 6 months in living donor kidney transplant recipients.

Registered in Clinical Trials Registry of India (CTRI/2011/091/000034)

## Introduction

Over the last 3 decades, calcineurin inhibitors (CNI) have been the mainstay of post-transplant immunosuppression. The improvement in short-term renal allograft survival seen with these agents, however, has not translated into similar degree of prolongation in long-term survival [1]. Progressive deterioration of allograft function is multifactorial, with chronic CNI toxicity being an important contributor. Long-term CNI use is also associated with other adverse effects such as increased risk of hyperglycemia and malignancies [2], [3]. The introduction of other immunosuppressive drugs such as mycophenolate mofetil (MMF) and sirolimus (SIR) raised the hope that elimination of CNI exposure might be possible [4], [5].

SIR, an inhibitor of the mammalian target of rapamycin (mTOR), was specially promising in this regard. When combined with CNI, SIR use leads to worsening of renal function as a result of potentiated nephrotoxicity [6]. CNI avoidance using SIR with anti-CD25 antibody or anti-thymocyte globulin, MMF and steroids, has provided comparable 1-year patient and graft survival and similar incidence of acute rejection. This has, however, come at the price of increased risk of surgical complications including lymphocele and delayed wound healing [7]–[10]. Late conversion after patients already showed evidence of CNI nephrotoxicity has been disappointing, as shown in the CONVERT trial [11].

Deferred pre-emptive switch to SIR from CNI after the period of highest immunological risk, but before development of CNI-related irreversible tubulointerstitial damage, can be a promising strategy. This approach entails replacing CNI with SIR after the period of risk for wound complications has passed (2 weeks to 6 months post-transplant). Studies evaluating this approach have reported a variable gain of renal function with different adverse event rate [12]-[18].

CD4+CD25+ regulatory T cell (T_reg_) suppress immune responses to self and non-self antigens and play an important role in the development and maintenance of transplantation tolerance in experimental models [19]. Increased T_reg_ number and T_reg_ associated gene expression profiles have been found in cell lines derived from renal transplant recipients with stable graft function compared with those with chronic allograft dysfunction [20].

SIR promotes conversion of CD4^+^CD25^naive^ T Cells to CD4^+^Foxp3^+^ T_reg_s [21]. In contrast, cyclosporine A (CsA) completely inhibits this process [22]. Therefore, use of mTOR inhibitors can help in achieving a state of relative immune tolerance by promoting T_reg_.

This study was done to evaluate the effectiveness of a deferred pre-emptive switch from a CNI-based therapy to a SIR-based therapy with continued CNI-based therapy in terms of the effect on GFR and T_reg_ population in primary recipients of living donor renal allografts.

## Materials and Methods

The protocol for this trial and supporting CONSORT checklist are available as supporting information; see Checklist S1 and Protocol S1.

### Ethics statement

The Postgraduate Institute of Medical Education and Research (PGIMER) Institute Ethics Committee approved the study protocol, and all subjects provided written consent. The study was limited to adult subjects. The trial was registered on the Clinical Trials Registry of India (http://ctri.nic.in/Clinicaltrials/; CTRI/2011/091/000034).

This prospective open label randomized trial was conducted at the Nehru Hospital of the Postgraduate Institute of Medical Education and Research, Chandigarh. Renal allograft recipients with stable graft function were randomized to either switch over to SIR or continue CNI after at least two months of kidney transplantation. Randomization was done with the help of a computer generated Bernoulli random number table (without blocking), and allocation concealment was achieved by opaque sequentially numbered sealed envelopes. The study was conducted according to the principles of the Declaration of Helsinki between March 2011 and December 2012.

### Inclusion and exclusion criteria

Patients of either sex between the age of 18 to 65 years who had undergone first live donor renal transplantation at least 2 months prior to enrolment and were receiving CNI based triple drug maintenance immunosuppression were eligible for study. Patients were required to have stable serum creatinine ≤1.2 mg/dl and proteinuria <500 mg/day.

Patients who had acute rejection, delayed graft failure, or were unable to achieve serum creatinine ≤1.2 mg/dl were not included. Patients with active infection in last 30 days, significant liver disease (continuously elevated aspartate and/or alanine aminotransferase levels >3 times the upper value of normal range during the past 30 days), those with severe diarrhoea, vomiting, malabsorption or active peptic ulcer disease or those on any investigational drug upto 4 weeks prior to assessment of eligibility were excluded from study. Other exclusion criteria were: pregnancy or failure to use effective birth control method in women of childbearing age; leukopenia (white blood cell count <3000 cells/µL) or thrombocytopenia (platelets <10,000 cells/µl). All patients were required to have fasting total cholesterol less than 200 mg/dl and fasting triglyceride □ 300 mg/dl with or without treatment. Patients suffering from any malignancy were not included.

Patients who refused further participation (withdrawal of informed consent) or developed concomitant disease or exacerbation of background disease that made it unsafe for the patient to continue in the study (considered as an adverse event) or protocol deviation prevented further participation, were allowed to leave the study.

### Endpoints

Primary endpoint was renal function assessed by serum creatinine-based GFR estimation by the 4-variable MDRD formula at the end of six months. The secondary endpoints were T_reg_ population at 6 months, incidence of biopsy proven acute rejection, patient and graft survival, incidence of hyperlipidemia, new onset diabetes after transplantation (NODAT), hypertension and infections.

### Immunosuppresion regimens

All patients were on a triple drug regime containing a CNI, either tacrolimus (Tacrograf, Biocon) or CsA (Cyclophil ME, Biocon), along with MMF (Renodapt, Biocon) and prednisolone. The target tacrolimus (Tac) C_0_ was 8–10 ng/mL for first 3 months and 6–8 ng/mL thereafter. For CsA, C_0_ was 200–300 ng/mL till month 3, and 150–250 ng/mL thereafter. MMF was started at 2 g/day and adjusted according to tolerability. Prednisolone was started at 500 mg methylprednisolone on the day of surgery, tapered to 5 mg/day by 8–12 weeks. All patients received one single strength cotrimoxazole tablet daily.

Patients randomized to SIR were advised to stop CNI 12 hours prior to initiating SIR. SIR was initiated on a loading dose of 6 mg for 2 days followed by 2 mg daily in a single dose. Trough level was checked after 5 days. Thereafter the dose was adjusted to achieve a trough level of 8–15 ng/mL. Blood levels of Tac, CsA and SIR were measured by liquid chromatography. All other variables were assessed by standard laboratory methodologies. Patients were followed up weekly for 4 weeks, fortnightly for next 8 weeks and monthly thereafter. All unexplained episodes of graft dysfunction were investigated by biopsy. Acute rejection was treated as per standard protocol.

### T_reg_ population

FoxP3+ T regulatory cell population was analyzed at baseline, and at 1, 3 and 6 months after enrolment. 150 µl of whole blood was surface stained with anti-human allophycocyanin (APC) conjugated-CD4 antibodies and phycoerythrin (PE)-Cy7 conjugated-CD25 antibodies(BD Biosciences) for 15 min at room temperature. RBCs were lysed with FACS-lysing solution (BD Biosciences). After washing with 1X PBS, cells were fixed for 10 min in FoxP3-fixation buffer (BD Biosciences) followed by permeabilization in FACSperm buffer for 30 min. Intracellular staining was done with Alexa-flour 488 conjugated-FoxP3 antibodies (BD Biosciences) for 30 min. Cells were washed, resuspended in PBS and analyzed on BD FACS Aria II (BD Biosciences). A total of 20000 events were acquired. CD25+ FoxP3+T cells were counted using FACS DIVA 6.0.

### Statistical analysis

The sample size was calculated to detect a mean change in estimated glomerular filtration rate (eGFR) of 8 mL/min (SD 10 mL/min). A sample size of at least 25 patients per treatment arm will provide at least 80% power at the 5% level.

Continuous variables are presented as mean ± SD and categorical variables as percentages. All categorical variables were analyzed by Chi square test or Fisher exact test as applicable. For between group comparisons, Student's T test was used if data was normally distributed, otherwise Mann Whitney test was used. For analyzing related variables within group, paired t-test was used. For more than two visits, comparison was done by analysis of variance (ANOVA) test. Analysis of covariance was done to adjust for other variables. A two-tailed p value of <0.05 was considered significant.

## Results

**Figure 1:**
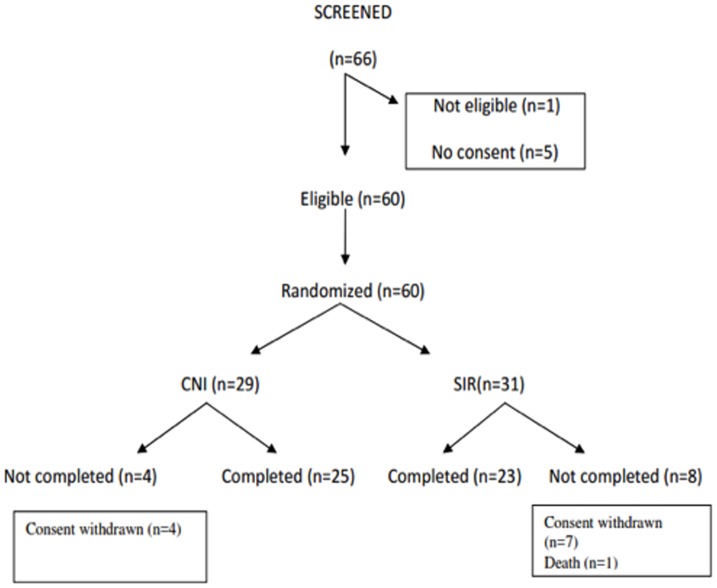
Study design, randomization, and follow up.

Out of a total of 66 patients screened, 60 fulfilled the inclusion and exclusion criteria and were randomized. 48 patients completed the study and were included in endpoint analysis (Fig. 1).

Table 1 gives the baseline characteristics of the study groups. More patients in CNI group had a genetically related donor, but other baseline parameters were similar in the two groups. Only 18.3% of the study population had received induction therapy. Median time of switch in both the groups was 2 months. Three patients in CNI group and 5 patients in SIR group were switched beyond 6 months after transplant.

**Table 1:** Baseline characteristics of study subjects in the two groups.

Parameter	Calcineurin inhibitor	Sirolimus	p value
**Number of cases**	29	31	
**Age (years)**	30.17±9.06	34.71±8.54	0.051
**Gender ratio M: F**	25∶4	27∶4	1.00
**Cause of ESRD**			
Unknown	26(89.7)	21(67.7)	0.242
Diabetic nephropathy	1(3.4)	5(16.1)	
Renal Stone Disease	1(3.4)	2(6.5)	
Vesicoureteric reflux	1(3.4)	1(3.2)	
ADPKD	0	2(6.5)	
**Donor age (years)**	42.00±9.05	39.06±8.73	0.206
**Donor-recipient relationship**			
First degree relative	22(75.9)	11(35.5)	0.002
Spouse/unrelated	7(24.1)	20(64.5)	
**Induction use**	3(10.3)	8(25.8)	0.184
**Type of CNI**			1
CsA	2	4	
Tac	27	27	
**CNI trough level (ng/mL)**			
CsA	300.94±5.14	280.95±79.94	1.00
Tac	13.46±3.98	12.92±5.05	0.418
**Time since transplantation (months)**	3.47±3.64	5.50±7.71	0.202
**Late conversions (>6 months)**	3	5	0.708
**New onset diabetes**	9(31.0)	7(22.6)	0.459
**Hypertension**	26(89.6)	27(87.1)	1.00

ADPKD: Autosomal dominant polycystic kidney disease, CNI: Calcineurin inhibitor, CsA: cyclosporine A, Tac: Tacrolimus.

Figures in parentheses are percentages.

About 90% of the study population was on Tac at the time of randomization (Table 1). Table 2 shows the achieved CNI and SIR level at different time points after randomization, which were within the desired target range. The groups had no difference in terms of BP control either at baseline or at end of study.

**Table 2:** Calcineurin inhibitor and Sirolimus trough levels at different time points.

	Days after randomization				
	7	30	60	120	180
Tacrolimus	10.26±3.18	8.22±02.23	7.60±2.12	7.18±2.09	6.70±2.05
Cyclosporine	260.94±5.14	194.96±23.75	171±25.77	159.45±23.58	154.02±5.86
Sirolimus	9.12±1.66	8.68±1.00	8.60±0.84	8.14±0.80	7.80±0.69

All values in ng/mL.

### Estimated GFR

**Figure 2:**
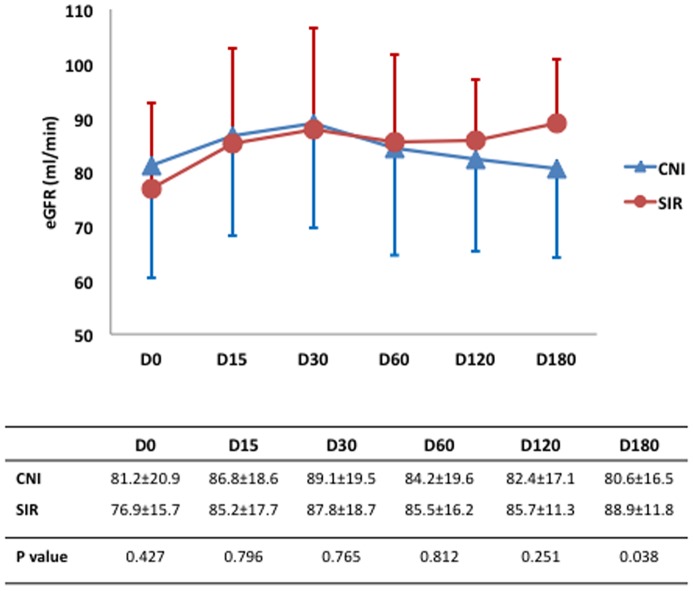
Graph showing estimated glomerular filtration rate (mL/min/1.73 m^2^) at different time points in the two groups. Patients in the SIR group showed a significant improvement in GFR over baseline, and the difference between groups was significant at 6 months.

There was no difference in eGFR between the two groups at baseline. At day 180, the eGFR in SIR group was 8.34 mL/min higher than CNI group (p = 0.038). After adjusting for age, the difference was even more significant (p = 0.002). Patients in the SIR group had a mean gain of eGFR of 12 ml/min (p = 0.040) whereas those in the CNI group showed no change (p = 1.0) (fig 2).

### T_reg_ population

**Figure 3:**
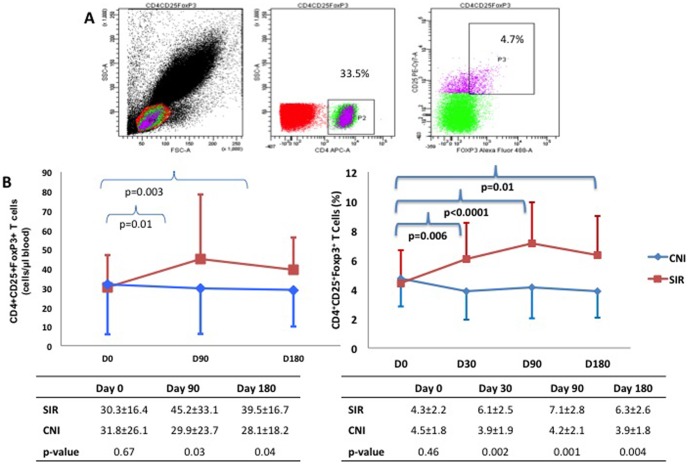
A. Dot plot showing analysis of (T_reg_) by flowcytometry and B. Graph showing T_reg_ number and frequency at different time points in the study in the two groups. Compared to baseline, SIR group showed higher T_reg_ number and frequency at all time points whereas there was no change in the CNI group. Compared to CNI group, SIR group showed higher T_reg_ number and frequency.

There was no difference in basal counts or proportions of T_reg_ (CD4+CD25+FoxP3+) in the two groups. The T_reg_ population showed a rise in the SIR group whereas it showed no change in the CNI group (fig 3). The difference between groups was significant at 30, 90 and 180 days. No correlation, however, was seen between T_reg_ number or percentage and eGFR.

### Adverse events

Two patients, both in the CNI group, had acute graft dysfunction. Both were diagnosed as acute rejection on graft biopsy: one patient had acute cellular rejection (ACR) while second patient had antibody mediated rejection (AMR). The ACR was steroid non-responsive; patient was treated with antithymocyte globulin, which produced only partial response. AMR was treated by plasma exchange, IV immunoglobulin and bortezomib, but the patient lost his graft and was back on dialysis. Both patients were excluded from analysis of primary outcome and T_reg_ analysis.

One patient each in both groups had respiratory tract infection. Skin infection, tuberculosis, herpes simplex virus infection was seen in one patient each, all in the CNI group. Herpes zoster was encountered in one patient in SIR group. No patient needed hospitalization. No patient had more than one infection during study period. None of the patients in either group developed genitourinary tract, gastrointestinal tract, central nervous system (CNS) or cardiovascular infection. None of the patient had cytomegalovirus or BK virus infection. One patient developed fulminant bacterial pneumonia one day after randomization to SIR arm. He could not recover despite treatment and expired.

Enthesitis was observed in 4 (17.39%) patients in SIR group. It was centered on the ankle joint in all cases. It responded to non-opoid analgesics and was self-limiting. None of the patients had to change the treatment because of enthesitis. No patient taking CNI had similar problem. One patient in SIR group, who was on antiepileptic treatment before randomization had breakthrough seizure despite adequate antiepileptic drug levels. Antiepileptic dose was increased. One patient in SIR group had aphthous stomatitis that improved spontaneously.

The two groups were similar in terms of their hematological parameters, lipid levels, liver function, and blood sugar profile at the end of the study (Table 3). A total of 9 (31%) patients in CNI group and 7 (22.6%) in SIR group had NODAT at the time of randomization. All patients maintained good glycemic control without any change in anti-diabetic treatment in both the groups. None of the patients developed proteinuria or NODAT after randomization.

**Table 3:** Blood and urine chemistries in the two groups at baseline and at day 180.

	Calcineurin inhibitor			Sirolimus			p value***
Parameter	Day 0	Day 180	p value*	Day 0	Day 180	p value**	
Serum creatinine (mg/dL)	1.16±0.28	1.14 ±0.17	0.580	1.14 ±0.17	0.99±0.11	0.001	0.773
Hb (g/dl)	12.04 ±1.57	12.12±2.02	0.897	12.33 ±1.70	11.85±1.64	0.374	0.543
TLC (/mm^3^)	7029±1312	6564±1091	0.110	7516±1444	6896±1193	0.088	0.227
Platelets (x10^5^/mm^3^)	3.05 ±0.97	2.20±0.68	0.340	2.77 ±0.99	2.63±0.66	0.579	0.321
ALT (IU/L)	27.29±13.48	24.28±13.42	0.075	31.38±16.10	26.54±6.96	0.075	0.779
AST(IU/L)	33.74±12.30	31.02±11.49	0.091	26.37±8.19	24.26±8.61	0.172	0.570
TC (mg/dl)	156.18±20.32	157.28±19.39	0.783	158.41±27.32	156.95±20.54	0.835	0.749
TG (mg/dl)	135.46±32.02	134.28±29.67	0.864	140.63±27.53	137.05±22.71	0.575	0.554
FPG (mg/dl)	98.02±11.60	103.16±7.18	0.072	98.59±18.26	104.57±6.24	0.183	0.898
Proteinuria (g/day)	110.31±28.42	95.92±21.80	0.064	102.83±28.72	94.71±20.35	0.295	0.369

Hb: Hemoglobin, AST: Aspartate aminotransferase; ALT: Alanine aminotransferase; TC: Total cholesterol; TG: triglyceride; FPG: Fasting plasma glucose.

* CNI Day 0 vs Day180. ^**^SIR Day 0 vs Day180.^***^At baseline, CNI vs SIR.

## Discussion

In this randomized controlled trial, we show that a deferred pre-emptive switch 2 months after transplantation from a CNI-based immunosuppressive regime to a SIR based one leads to a significant improvement in GFR over 6 months without a side effect penalty. Furthermore, we show that change to SIR results in a consistent and sustained expansion in the regulatory T-cell population.

We randomized the patients 2 months post-transplant in order to avoid the period of maximum immunological risk. At that time, the chances of surgical problems are likely to be minimal and exposure to CNI will not be that advanced that attempt to preserve renal function might be futile. A small number (3 in CNI group and 5 in SIR) were randomized six months after transplantation. However, number of such patients was rather small and they were equally distributed in the two groups. Thus our randomization style was similar to that of CONCEPT Trial [17].

In terms of primary end point, our findings confirmed the observational studies and clinical trials that show better preservation of GFR with mTOR inhibitors compared to CNIs [12]–[18]. There was gain of eGFR of 8.23 ml/min as early as day 15 in SIR group, which had increased to 12 ml/min at 180 days. In contrast, the eGFR did not change in the CNI group. In our final analysis of eGFR, we did not include data of the patients who had acute rejection. Both the patients were in CNI group. If we had included these two patients, the difference in eGFR would have been even more robust. This gives further strength to our results.

In contrast to other studies that have shown a high incidence of side effects after conversion to SIR, we noticed surprisingly small number of adverse events and none of the patients needed to discontinue SIR due to adverse events. There was no episode of rejection in SIR group and only two rejections in CNI group. Both the patients could not achieve baseline renal function and one patient had graft loss. BPAR in patients taking SIR at 12 months in SMART [14], CONCEPT [17], and CONVERT [11] trials has been found to be around 17%, whereas it was 7.4% in Spare the Nephron trial.[18] The infection rates in SIR and CNI groups (17% vs 8.5%) were no different, a finding similar to the SMART, CONCEPT and Spare the Nephron trials. In CONVERT trial, however, infection rates were higher in patients taking SIR. Another encouraging finding was lack of development of proteinuria in the SIR group. Proteinuria >1 gm was seen in only 5.8% and 4.2% patients at the end of 2 and 3 years respectively in the Zeus trial. Likewise, in Spare the Nephron trial only 3 patients had to switch treatment for proteinuria and in CONCEPT trial it was less than 10%. However, trials with late conversion like CONVERT trial showed proteinuria of 23.6%. As proteinuria is considered to be a marker of health of graft kidney over long term, this distinction of early and late conversion is of massive importance [11], [13], [17], [18].

At the end of six months, hemoglobin and lipid levels in our study were not significantly different in both the groups from baseline. Spare the Nephron[18] and CONVERT[11] trials reported a higher incidence of anemia in patients taking SIR, but incidence was similar in SMART[14] and CONCEPT[17] trials. Similarly, the effect on lipid profile is inconsistent across studies [11], [14], [16]–[18].

Mouth ulcers have been reported as a consistent bothersome adverse effect in 20–46% patients using SIR. In our study, however, only one patient complained of aphthous stomatitis. Since this problem is encountered early after exposure to SIR, we hope that this excellent tolerability will be maintained over long term as well [11], [14]–[18].

One unique adverse event in patients taking SIR was enthesitis, not previously reported with SIR. This suggests the need of vigilance for musculoskeletal side effects on further follow up.

No patient refused further participation because of any side effect. Patients who withdrew consent, they did it in early part of study. At that time more frequent visits were required, that they were not able to follow-up.

There are some differences in the patient population in this study from the ones reported earlier. We included only patients with live donor first time kidney transplant. Only 18% of our study population received induction therapy. In SMART[14], ZEUS[12] and CONCEPT[17] studies, all patients received induction therapy while in SPARE THE NEPHRON[18] trial 70% of the study population had received induction therapy. These trials included deceased donor renal transplants as well. Thus risk profile of our patients should be seen in the context of less robust induction therapy as well.

Search is on for strategies that could affect the repertoire of immune cells in a way as to tilt the balance in favor of cells that would inhibit antigen specific response. CD4+CD25+ T regulatory cell population is thought to be instrumental in downregulating alloantigen specific immune response. T_reg_ generation and maintenance requires activation by T cell receptor engagement and IL-2 signaling. However, the influence of immunosuppressive medications on T_reg_ homeostasis in humans *in vivo* remains a subject of exploration.

We found prompt and sustained expansion in the T_reg_ population with SIR but not CNI. This effect was be not affected by prior exposure to CNIs. In an observational study, T_reg_ frequency in peripheral blood of renal transplant patients receiving CNI was lower compared to those receiving SIR [23]. Korczak-Kowalska showed that T_reg_ percentage in SIR treated patients did not differ from that observed in healthy individuals, but was significantly higher compared with CsA-treated patients [24]. This effect seems to be unaffected by the duration of transplant. In a recent study, Carroll et al [25] showed similar increase in T_reg_ population in 13 renal transplant recipients with squamous cell carcinoma randomized to SIR 21 years after transplantation. This expansion was not noted in the comparator CNI arm. A similar increase has been seen following induction with Campath-1H [26]. Noris et al showed that the reconstituted T-cell population following depleting induction using Campath-1H had greater proportion of T_reg_ in patients who received SIR compared to those on CsA [27]. It can be speculated, therefore, that a combination of Campath-1H induction and maintenance therapy with SIR may be tolerogenic. The long-term sustainability of this expansion needs to be evaluated. We also need to study the differential effect of these two agents on other immune parameters such as B-cell division and dendritic cell function.

This trial had certain limitations. The CNI group had larger proportion of genetically related donors. Because of resource limitations, HLA matching was not performed for genetically unrelated donors (including spouses), but the CNI group had potentially greater degree of HLA matching. Another caveat that limits the applicability of our results is the short duration of follow-up. These patients will need to be observed for a longer period to confirm these findings.

In conclusion, deferred preemptive conversion from CNI to SIR 2 months after transplant in low risk recipients is safe and leads to a gain of about 12 mL/min over a 6-month period. Switch to SIR is also associated with expansion in T_reg_ population. These findings need confirmation on longer follow up.

## Supporting Information

Checklist S1 **Supporting CONSORT checklist.**
(DOC)

Protocol S1 **Trial protocol.**
(PDF)
