# Supplementary material for: Deferred Pre-Emptive Switch from Calcineurin Inhibitor to Sirolimus Leads to Improvement in GFR and Expansion of T Regulatory Cell Population: A Randomized, Controlled Trial
Source: PLoS One. 2013 Oct 11;8(10):e75591. doi: 10.1371/journal.pone.0075591 (PMC3795731; doi:10.1371/journal.pone.0075591)
Supplement: Protocol S1 — Trial protocol. (PDF) [file pone.0075591.s002.pdf]

AN OPEN-LABEL, RANDOMIZED, PROSPECTIVE STUDY TO EVALUATE THE EFFICACY AND SAFETY OF CONVERSION TO SIROLIMUS BASED REGIMEN FROM CALCINEURIN INHIBITORS BASED REGIMEN IN MAINTENANCE KIDNEY TRANSPLANT RECIPIENTS

INTRODUCTION

Since the early 1980s, the standard approach to immunosuppression in transplant recipients has involved the use of calcineurin inhibitors (CNI) such as cyclosporine (CsA) and tacrolimus. CNIs reduce the number of acute rejection episodes and enhance short-term allograft survival.[1] However, the greater benefit appears to be a decrease in the number of acute rejections during the first months after transplantation. Progressive chronic nephrotoxicity is a major toxic effect of CNI in the long term and is associated with mild-to-moderate renal dysfunction. Death from cancer or cardiovascular diseases with a functioning graft is also a major cause of late graft loss.[2,3] The balance between preventing immunological allograft failures, avoiding over immunosuppression and managing nephrotoxicity is therefore still an unresolved issue. The introduction of immunosuppressive drugs such as mycophenolate mofetil (MMF) and sirolimus (SRL) has strengthened the case for minimizing the use of CsA.[4,5] SRL is an antifungal macrolide that displays potent antiproliferative activities that produce immunosuppressive effects. Binding of SRL to the intracellular immunophilin FKBP12 blocks the activity of the mammalian target of rapamycin (mTOR) with potent inhibition of downstream signal ligand progression from the G1 to the S phase of the cell-cycle. SRL has been shown to reduce the incidence of acute rejection after renal transplantation, without appearing to cause significant inherent nephrotoxicity in most animal and human studies.[6] However, when combined with CNI therapy, renal function often worsens as a result of potentiated nephrotoxicity.[7] SRL has therefore been used as a primary immunosuppressant in CNI-free regimens. Together with anti-CD25 antibody or anti-thymocyte globulins, MMF and steroids, these regimens have provided comparable 1-year patient and graft survival, and incidence of acute rejection episodes.[8,9] Furthermore, renal function was also significantly better in the CsA-free group and did not tend to worsen during the first year after transplantation. However, early use of SRL after transplantation has been demonstrated to be associated with various complications including lymphocele, prolonged delayed graft function (DGF), delayed wound healing and by a slight increased incidence of acute rejection.[9,10] This study could help in evaluating the ideal dose, trough blood level, safety and benefits of conversion to Sirolimus based regimen from CNI based regimen in Indian patients.

REVIEW OF LITERATURE

During the past decade there has been a major shift in the focus of kidney transplant research from the prevention of acute rejection to the recognition and targeting of risk factors that associate with late graft loss beyond 1 year. It is now apparent that the major causes of kidney graft loss beyond 1 year after transplantation are progressive deterioration of graft function, often termed chronic allograft nephropathy (CAN), the hallmark of which is histological evidence of tubular atrophy, interstitial fibrosis, and transplant glomerulopathy; and patient death from cardiovascular disease, often with a functioning kidney graft. What has also emerged is that many of the same risk factors associated with the former are also associated with the latter. These include age, diabetes, both preexisting and occurring after the transplantation, poorly controlled hypertension, dyslipidemia, viral infections, and diminished allograft function. In fact, renal dysfunction (estimated glomerular filtration rate (eGFR) <40 cc/min) itself appears to be an independent risk factor for coronary heart disease and death.[11]

A cruel irony that has accompanied the control of acute rejection via the widespread introduction of CNI drugs nearly 30 years ago has been their direct and indirect toxicity to the kidney. CNI nephrotoxicity appears to target the renal microcirculation by promoting preglomerular vasoconstriction, as well as direct injury resulting in hyalinosis of blood vessels. In addition, CNIs appear to favor the progression of post-transplantation hypertension, hyperglycemia, dyslipidemia, and oncogenesis, which are associated with late morbidity. Although variable in severity and often dose related, CNI nephrotoxicity contributes to the decline in kidney function over time.[3] The pattern of injury is perhaps best chronicled in non-transplant patients treated for autoimmune disease who lost 50% of their native renal function and developed histological striped fibrosis after 2 years of continuous CNI use.[12]

For these reasons, CNI dose reduction, elimination, or avoidance has become the focus of numerous clinical kidney transplant trials with measurements of renal function rather than acute rejection as the primary efficacy end point.

In fact, initial experiences of CNI avoidance in a single-arm study with MMF, daclizumab and steroids resulted in unacceptably high acute rejection rates, though with good renal

function and patient and graft survival.[13] SRL, a potent macrolide immunosuppressant claimed as a non-nephrotoxic drug, was initially tested in clinical trials in association with CNIs. This combination greatly reduced acute rejection rates, but resulted in increased nephrotoxicity.[14,15] Thus, the CNIs-SRL combination is not so far the ideal immunosuppression for avoiding nephrotoxicity in renal transplantation.

Some clinical investigators combined SRL with MMF in completely CNI-free protocols and it has been demonstrated by comparison of 2-year kidney biopsies that recipients treated with mycophenolate mofetil (MMF) and steroids and the mTOR inhibitor sirolimus in lieu of a CNI drug had diminished scarring and gene expression for fibrosis and tissue remodeling.[6]

Synergistic action of MMF and SRL in prevention of chronic allograft damage

MMF is a reversible inhibitor of inosine monophosphate dehydrogenase (IMPDH), blocking proliferation of lymphocyte[16]; and SRL blocks cell proliferation induced by growth factors and cytokines by inhibiting the signaling protein mTOR (mammalian target of rapamycin)[17]. Furthermore, it has been described that the use of MMF in conjunction with SRL reverses its effect on transforming growth factor-β production without apparent nephrotoxicity.[18]All these facts suggest that the association of these two drugs may be useful in the design of non-nephrotoxic immunosuppressive regimens, and potent enough to keep down the acute rejection rate (Figure 1).

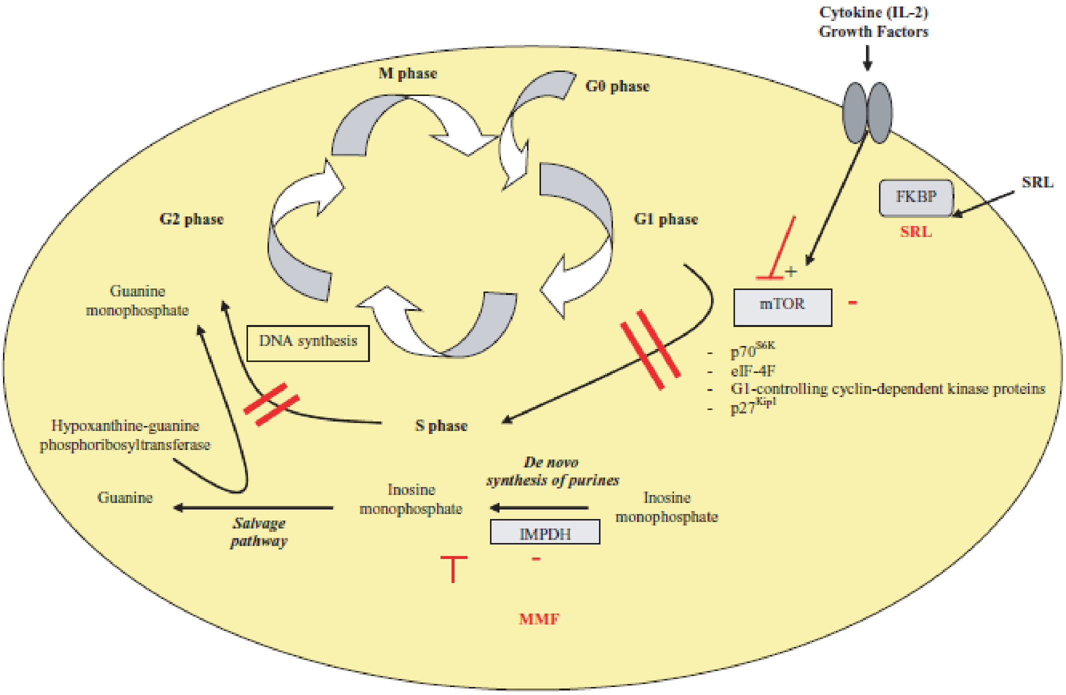

Figure 1: Synergistic action of MMF and SRL

In graft vasculopathy, abnormal vascular SMC proliferation and migration play major roles. In normal vessels, most SMC reside in the media, in which they are quiescent and possess a contractile phenotype characterized by the abundance of actin- and myosin-containing filaments. In disease states, SMCs re-enter the cell cycle, proliferate and migrate from the media to the intima. SRL inhibits both human and rat SMC proliferation by blocking cell-cycle progression at the G1/S transition and blocks rat, porcine and human SMC migration.[19] In a nonhuman primate model of allograft vascular disease, the delayed introduction of SRL, to allow the development of vascular disease, halted the progression of pre-existing intimal thickening and was also associated with partial regression. The size of the allograft intimal area correlated inversely with SRL trough levels. In the same model, treatment with SRL from the day of transplantation prevented graft vascular disease.[20] MMF was also studied for the prevention of allograft vascular disease in a similar experimental model.[21] Although in this study there was no overall statistically significant difference in progression of graft vascular disease between the untreated and MMF-treated animals, there was close correlation between the total daily MMF dose and intimal hyperplasia. Depending on the individual tolerability of MMF in cynomolgus monkeys, MMF was effective for treatment of advanced graft vasculopathy after aortic transplantation. This efficacy was achieved when intimal proliferation had already proceeded unhindered for more than 6 weeks after transplantation due to lack of any immunosuppression. In a previous study in rodents, MMF and SRL more effectively inhibited intimal thickening than either drug alone[22], suggesting that the combined therapy of MMF and SRL may be effective for controlling graft vasculopathy in nonhuman primates or patients. In a classic model of chronic allograft rejection in rats, the effects of these two immunosuppressants were evaluated in a Lewis-to-Fisher donor/recipient strain combination.[23] Rats treated with MMF or SRL alone showed a Banff sum score similar to the allograft control group. When the recipient rats were treated with MMF and SRL in combination, there was a statistically significant reduction of Banff sum score, with specific inhibition of vascular fibrous intimal thickening, allograft glomerulopathy and interstitial fibrosis. All these experimental studies suggest that the combination of MMF and SRL might be relevant to the prevention of acute rejection and CAN, especially graft vasculopathy.

Acute rejection prophylaxis: non CNIs based regimens

De novo CNIs avoidance

In a trial comparing corticosteroids, SRL and MMF versus corticosteroids, CsA and MMF[24], the incidence of biopsy-proven acute rejection at 12 months was not statistically significant

(27.5% in SRL and 18.4% in CsA), but in this study nearly 50% of patients treated with SRL and MMF received bolus steroids. They found a great number of adverse events in the SRL group, especially diarrhea, thrombocytopenia, hypercholesterolemia, hypertriglyceridemia and infectious pneumonia. However, dose of SRL and MMF dose was much higher than those now recommended. Indeed, target trough levels of SRL were 30 ng/mL for 2 months and 15 ng/mL thereafter, and MMF was given at 2 g a day. In an attempt to improve efficacy, Flechner et al. added induction therapy with basiliximab. The 2-year graft survival was greater than 90%, and the incidence of biopsy-proven acute rejection was 6.5% in SRL and 16.6% in CsA.[6,25] Similar results were found when polyclonal antilymphocyte antibodies were used as induction therapy. In a randomized study of 132 living-donor renal recipients, Hamdy et al. compared SRL with low-dose TAC versus SRL-MMF in patients all receiving basiliximab and steroids. Incidence of acute rejection was 18.5% in SRL-TAC versus 13.5% in SRL-MMF.[26]

The efficacy of the SRL and MMF combination for the prevention of acute rejection in a steroid-free protocol was also tested using Alemtuzumab induction therapy.[27]In this study, the incidence of acute rejection was 36.3%.

The de novo use of lower-dose SRL (trough target range, 4–8 mg/ml) in combination with MMF has resulted in an increased risk of acute rejection, treatment failure, and reduced allograft survival compared with MMF/ tacrolimus[28] and the use of SRL in early post surgical period has been variably associated with problems of wound healing.[24,25,31]

Switch to SRL in maintenance phase

In recently published CONCEPT study, patients were converted to SRL at 3 months or maintained on cyclosporine, both in combination with MMF. In this study, patient and graft survival were not significantly different, but patients on the SRL regimen had improved renal function at the end of 1 year.[29] The CONVERT trial findings indicated that improved graft function occurs in patients with eGFRs above 40 ml/min and a urine protein:creatinine ratio < 0.1 when they are randomly switched from CNIs to SRL 6 to 120 months post transplant period.[30] In recently published Spare the Nephron trial, random switch over to SRL from CNIs 30 to 180 days post transplant resulted in similar measures of renal function but with fewer deaths and a trend to less Biopsy proven acute rejection and graft loss over 2 years of study period.[31]

Renal function

Studies have shown that early after transplantation, glomerular filtration rate (GFR) was consistently higher in patients receiving SRL-MMF than CNIs-MMF.[24,25] However, recently, Larson et al. reported that the iothalamate clearance at 1 month was higher in the SRL-MMF than in the TAC-MMF group (67 ± 18 mL/min vs. 58 ± 17 mL/min). But, this difference was lost at 1 year due to unexpected loss of GFR in the SRL-MMF group.[32]

CAN

Flechner et al. showed that at 2 years a third of patients treated with SRL-MMF had normal biopsies while 79.2% of patients treated with CsA-MMF had histological evidence of CAN.[27] Moreover, Larson et al. observed that, although renal function was similar, chronic vascular damage was significantly lower in the SRL-MMF than in the TAC-MMF combination (26% vs. 43%).[32] Thus, the SRL-MMF combination as maintenance immunosuppression is associated with a reduction in chronic renal damage in the majority of studies performing protocol biopsies. However, whether histological benefit depends on avoiding CNIs or arises from the direct antifibrotic effect of the SRL-MMF combination is not known.

Adverse events

SRL and MMF are potent immunosuppressants that have antiproliferative properties and interfere at different steps of the cellular cycle. Each drug is associated with a particular profile of side effects; some of them could be amplified or minimized when SRL and MMF are co-administered (Figure 2).

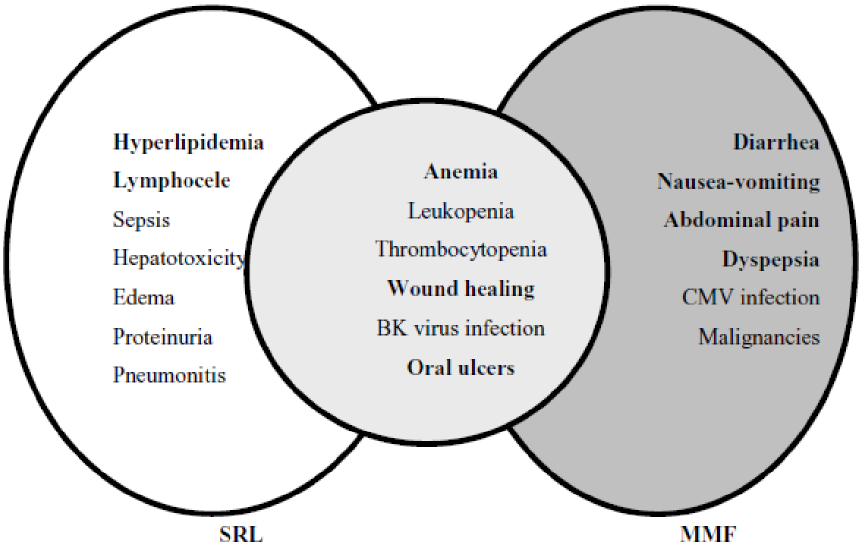

Figure 2: Adverse events in SRL-MMF combination

Bone marrow suppression: Both drugs are associated with bone marrow suppression, in fact, in both SRL- and MMF-treated patients, the incidence of leukopenia (leukocytes < 3000 per mm^3) and thrombocytopenia (platelets < 90000 per mm^3) was 20–45%.[24,25] Anemia is perhaps the paradigm of adverse event amplification. Anemia is increasingly recognized as an important posttransplant comorbidity. Posttransplant anemia has multifactorial etiology, such as poor graft function, iron deficiency and some antihypertensive and immunosuppressive medications. MMF by its inherent mechanism of action was early recognized as a potent bone marrow suppressor and is considered an independent predictor of posttransplant anemia, which seems to be dose-dependent. More obscure and ill-defined are the mechanisms of SRL-related anemia. In a recent paper, Augustine et al. showed a clear association between posttransplant anemia and SRL (57% of SRL-treated patients had anemia 12 months after transplantation). The authors postulate that SRL induces an erythropoietin-resistance state by inhibiting erythropoiesis at the level of the erythropoietin receptor, since the mTOR downstream pathway is critical for erythroid cell replication.[33] This feature may be important after transplantation, when the patient needs to recover from postsurgical anemia.

Gastrointestinal side effects: SRL and MMF pharmacokinetic interaction: These adverse events are mostly related to MMF toxicity although SRL was also described as a cause of intestinal ulcerations and diarrhoea. In fact, painful oral ulcers are frequently reported in patients treated with SRL-MMF.[34] On the other hand, Kreis et al. reported higher incidence of diarrhoea in SRL-MMF than in CsA-MMF (38% vs. 11%).[24] Interestingly, these authors' pharmacokinetic studies showed that, with an equivalent MMF dose, MPA trough levels were significantly higher in SRL- than in CsA-treated patients. Elsewhere, a pharmacokinetic explanation of these findings was provided: that CsA decreases biliary excretion of MPA glucuronide by a mechanism that involves inhibition of multidrug resistance-associated protein 2, blocking an important mechanism for maintaining MPA concentration.[35] In a recent paper, Cattaneo et al. found that SRL but not CsA treated patients showed a second peak consistent with the enterohepatic recirculation of MPA. The result is that nearly half a dose of MMF is usually required to maintain adequate MPA concentration in CsA-free regimes.[36]However, it should be pointed out that the value of trough MPA concentration for MMF therapy still has to be determined.[35]

Lymphocele and wound-healing complications: The use of SRL and MMF has been described as causing cicatrization problems. This kind of adverse event is poorly defined in most studies and its incidence could range between 5% and 40%.[24,25] However, all immunosuppressive agents inhibit to some extent wound healing, especially steroids Although controversial, these results suggest that in the SRL-MMF combination, experience is critical in making refinements that lead to a significant improvement in the postsurgical period. All these observations raise the question of when to start SRL after transplant surgery.

MATERIAL AND METHODS

PATIENTS

Total number of patients to be studied will be 60.

INCLUSION CRITERIA

- Willing to sign informed consent
- Adult renal transplant recipients of either sex over the age of 18 years
- Undergone denovo live donor renal transplantation at least 2 months prior to enrolment
- Receiving CNI based maintenance regimen after transplantation
- Serum creatinine ≤1.2mg/dl and proteinuria <800mg/day

EXCLUSION CRITERIA

- Pregnant, nursing mothers or women of childbearing potential without an effective method of birth control.
- Renal transplant recipients who had episodes of acute rejection, delayed graft failure or signs of graft failure and were unable to achieve serum creatinine ≤1.2mg/dL.
- Patients suffering from any malignancy requiring immediate surgery, ongoing chemotherapy or radiation.
- Patients suffering from any acute systemic infection within 30 days prior to enrolment.
- Use of any investigational drug upto 4 weeks prior to enrolling in the study.
- Patients requiring second renal transplantation.
- Patients with untreated hypercholesterolemia defined as having total cholesterol >200 mg/dl, triglycerides > 300mg/dL.

- Patient is leukopenic defined as WBC count < 3000 cells/μL or thrombocytopenic defined as platelets < 10000 cells/μL.
- Patient has significant liver disease defined as having during the past 30 days continuously elevated SGOT and or SGPT levels greater than 3 times the upper value of normal range.
- Patients suffering from severe diarrhoea, vomiting, GI malabsorption or active peptic ulcer disease.

Withdrawal from study

1. Refuse further participation (withdrawal of informed consent).
2. Concomitant disease or exacerbation of background disease that may be unsafe for the patient to continue in the study (considered as an adverse event).
3. Protocol deviation that would prevent further participation in the study
4. Lost to follow-up.

STUDY METHOD

This will be an open label, prospective, randomized, two arm study to be conducted in renal transplant recipients who have undergone renal transplant >2 months prior to enrollment. Renal transplant recipients who had undergone live donor renal transplantation and are on CNI based maintenance regimen will be screened for inclusion and exclusion criteria. Informed consent will be taken after explaining the objectives and procedures of the trial from all the patients who agree to participate in the study. Subjects who satisfy all inclusion and exclusion criteria will either continue with existing CNI regimen or converted from CNI based regimen to sirolimus with CNI withdrawal.

At baseline patient's past, family, medical, transplant and medication history will be enquired and relevant findings will be recorded in the CRF. Patient's general and physical findings will be recorded. Baseline laboratory investigations including CBC, electrolytes, fasting blood sugar, renal profile, SGOT, SGPT, Lipid profile, urine analysis and 24 hrs urine test for urine proteins will be done and recorded. Baseline and subsequent GFR will be calculated by MDRD method. CNI dose and blood levels of all the patients will be recorded at baseline. Subsequently the patients will be randomized either to CNI group who will continue with existing dose or to Sirolimus group. Patients allocated to Sirolimus group, will be initiated

with a loading dose of 6mg for 2 days and later the dose will be adjusted to achieve a blood level of 8-15ng/ml. The CNIs in sirolimus group will be discontinued 12 hrs prior to initiating Sirolimus. Later, in CNI or sirolimus group the dose of individual drugs will be titrated as per target blood level and clinical response. Concomitant immunosuppressants like Mycophenolate mofetil (MMF) and steroids will be continued as per the clinical practice. All the drugs for any pre-existing or co-existing illness will be allowed unless those drugs are contraindicated. All the subjects will be assessed at regular intervals and at the end of study period of 6 months. Relevant laboratory investigations will be done at defined intervals and all the investigations done at baseline will be repeated at the end of study period.

FoxP3+ T regulatory cell population will be analyzed at baseline, and at 1, 3 and 6 months after enrolment. By flowcytometery using anti-human allophycocyanin (APC) conjugated-CD4 antibodies, phycoerythrin (PE)-Cy7 conjugated-CD25 antibodies(BD Biosciences) and Alexa-flour 488 conjugated-FoxP3 antibodies (BD Biosciences) on BD FACS Aria II (BD Biosciences). A total of 20000 events will be acquired, and CD25+ FoxP3+T cells will be counted using FACS DIVA 6.0.

1: 1 randomization will be done with the help of a computer generated random number table, and allocation concealment will be achieved by opaque sequentially numbered sealed envelopes. The response of the patient to the therapy will be in terms of renal function assessment. The primary analysis will be two-sided independent sample t-test for difference of means for GFR estimation by MDRD method.

A sample size of at least 25 patients per treatment arm will provide at least 80% power based on the assumption of an effect size (difference of GFR estimates between two treatment arms) of 8 mL/min and S.D. as 10 mL/min

The secondary endpoints will be:

- Acute rejection
- Patient and graft survival at 6 months,
- Incidences of hyperlipidemia, NODM, hypertension and infectious complications
- Regulatory T-cell population

All patients will be followed up at 15, 30, 60, 120 and 180 days. All investigations required to determine the primary and secondary end points will be performed. Sirolimus levels will be determined at 5, 12, 30, 60 and 120 days in patients randomized to sirolimus arm whereas those on tacrolimus will have measurements at 30, 60 and 120 days.

The target trough levels are as follows:

- Sirolimus: 8-15ng/ml
- Tacrolimus: 8-10 ng/ml upto mo 3, 6-8 ng/ml thereafter
- Cyclosporine: 200-300 ng/ml upto mo 3, 150-250 ng/ml thereafter

All unexplained episodes of graft dysfunction will be investigated by biopsy. Acute rejection will be treated as per standard protocol.

Variables will be presented as mean ± SD and categorical variables as percentages. Appropriate statistical studies will be used for analysis of results. Two-tailed P-value <0.05 will be considered significant.

Support:

The study drugs (sirolimus, tacrolimus and cyclosporine) will be provided by Biocon India, Bangalore.

ANNEXURE 1

INFORMED CONSENT FORM

Date:

Study name: An open-label, randomized, prospective study to evaluate the efficacy and safety of conversion to sirolimus based regimen from calcineurin inhibitors based regimen in maintenance kidney transplant recipients

Researchers: Dr. Dinesh Bansal, senior Resident, Nephrology, PGIMER

Dr. Vivekanand Jha, Professor of Nephrology, PGIMER

Dr. V. Sakhuja, Professor and head Nephrology, PGIMER

Dr. Mukut Minz, Professor and head Transplant surgery, PGIMER

Sponsors: Postgraduate Institute of Medical Education and Research, Chandigarh

Purpose of the research: To compare the efficacy and safety of conversion to Sirolimus from calcineurin-inhibitor based therapy in maintenance renal transplant recipients

What you will be asked to do in the research: To take drugs, regular follow up and investigations as advised

Risks and discomforts: We do not foresee any risks or discomfort from your participation in the research.

Benefits of the research and benefits to you: As CNIs can result in loss of function of kidney in long term, sirolimus as replacement can be helpful to avoid this.

Voluntary participation: Your participation in the study is completely voluntary and you may choose to stop participating at any time. Your decision not to volunteer will not influence the treatment you may be receiving, or the nature of your relationship with PGIMER either now, or in the future.

Withdrawal from the study: You can stop participating in the study at any time, for any reason, if you so decide. If you decide to stop participating, you will still be eligible to receive the promised benefits for agreeing to be in the project. Your decision to stop participating, or to refuse to answer particular questions, will not affect your relationship with the researchers, PGIMER, or any other group associated with this project.

Confidentiality: Unless you choose otherwise, all information you supply during the research will be held in confidence and unless you specifically indicate your consent, your name will not appear in any report or publication of the research. Your data will be safely stored in a locked facility and only research staff will have access to this information. Confidentiality will be provided to the fullest extent possible by law.

Questions about the research? If you have questions about the research in general or about your role in the study, please feel free to contact Dr. Dinesh Bansal either by telephone at +919592088575, or by e-mail (mamcguy123@rediffmail.com). This research has been reviewed by the Institute Ethics Committee of the PGIMER and conforms to the standards of the Indian Council of Medical Research Ethics guidelines. If you have any questions about this process, or about your rights as a participant in the study, please contact Prof A Chakrabarty, Department of Medical Microbiology, PGIMER (telephone 0172-275-5156 or e-mail arunaloke@hotmail.com).

Legal Rights and Signatures:

I____, consent to participate in the study entitled "AN OPEN-LABEL, RANDOMIZED, PROSPECTIVE STUDY TO EVALUATE THE EFFICACY AND SAFETY OF CONVERSION TO SIROLIMUS BASED REGIMEN FROM CALCINEURIN INHIBITORS BASED REGIMEN IN MAINTENANCE KIDNEY TRANSPLANT RECIPIENTS "conducted by Dr.Dinesh Bansal. I have understood the nature of this project and wish to participate. I am not waiving any of my legal rights by signing this form. My signature below indicates my consent.

Signature ______________________ **Date**______________________________

Participant

Signature ______________________ **Date**______________________________

Principal Investigator

Signature ______________________ **Date**______________________________

Witness

STUDY VISIT PLAN

Visit	0	1	2	3	4	5
Days		0 - 14 (± 2days)	15- 30 (± 2days)	60 (± 7days)	120 (± 7days)	180 (± 7days)
Informed consent	X					
Medical/ Transplant history	X					
General exam	X					
Physical exam	X					
Vital Parameters (PR, BP, Fluid Intake and Urine output)	X	X	X	X	X	X
**Blood tests** CBC, Serum creatinine, Blood urea, electrolytes, Blood sugar, lipid profile, SGOT, SGPT, Urine for routine analysis, 24hrs urine for protein and Urine protein to creatinine ratio	X	---------	--------	--------	X	X
**Therapeutic drug monitoring**						
i. Rapacan group	-----	X	X	X	X	X
ii. CNI Group	-----	------	------	X	X	X
**Dose titration of**						
i. Rapacan		X	X	X	X	X
ii. CNI's		X	X	X	X	X
Dose adjusts. Of concomitant drugs		X	X	X	X	X
Recording of adverse effects		X	X	X	X	X

BIBLIOGRAPHY

1. The Canadian Multicentre Transplant Study Group. A randomized clinical trial of cyclosporine in cadaveric renal transplantation. Analysis at three years. *N Engl J Med* 1986; 314: 1219–1225.
2. Bennett WM, DeMattos A, Meyer MM, Andoh T, Barry JM. Chronic cyclosporine nephropathy: The Achilles' heel of immunosuppressive therapy. *Kidney Int* 1996; 50: 1089–1100.
3. Nankivell BJ, Borrows RJ, Fung CL, O'Connell PJ, Allen RD, Chapman JR. The natural history of chronic allograft nephropathy. *N Engl J Med* 2003; 349: 2326–2333.
4. Mourad G, Vela C, Ribstein J, Mimran A. Long-term improvement in renal function after cyclosporine reduction in renal transplant recipients with histologically proven chronic cyclosporine nephropathy. *Transplantation* 1998; 65: 661–667.
5. Weir MR, Blahut S, Drachenburg C *et al.* Late calcineurin inhibitor withdrawal as a strategy to prevent graft loss in patients with suboptimal kidney transplant function. *Am J Nephrol* 2004; 24: 379–386
6. Flechner SM, Kurian SM, Solez K *et al.* De novo kidney transplantation without use of calcineurin inhibitors preserves renal structure and function at two years. *Am J Transplant* 2004; 4: 1776–1785.
7. Meier-Kriesche HU, Steffen BJ, Chu AH *et al.* Sirolimus with neoral versus mycophelonate mofetil with neoral associated with decreased renal allograft survival. *Am J Transplant* 2004; 4: 2058–2066
8. Larson TS, Dean PG, Stegall MD *et al.* Complete avoidance of calcineurin inhibitors in renal transplantation: A randomized trial comparing sirolimus and tacrolimus. *Am J Transplant* 2006; 6: 514–522.
9. Buchler M, Caillard S, Barbier S *et al.* Sirolimus versus cyclosporine in kidney recipients receiving thymoglobulin, mycophenolate mofetil and a 6-month course of steroids. *Am J Transplant* 2007; 7: 2522–2531.
10. Valente JF, Hricik D, Weigel K *et al.* Comparison of sirolimus vs. mycophenolate mofetil on surgical complications and wound healing in adult kidney transplantation. *Am J Transplant* 2003; 3: 1128–1134.

11. Israni AK , Snyder J , Skeans M *et al.* Predicting coronary heart disease after kidney transplantation: patient outcomes in renal transplantation . *Am J Transplant* 2010; 10: 338 – 353.

12. Palestine AG, Austin H, Balow JE *et al.* Renal histopathologic alterations in patients treated with cyclosporine for uveitis . *N Engl J Med* 1986; 314: 1293–1298.

13. Vincenti F, Ramos E, Brattstrom C *et al*. Multicenter trial exploring calcineurin inhibitors avoidance in renal transplantation. Transplantation 2001; 7: 1282–1287.

14. McAlister VC, Gao Z, Peltekian K, Domingues J, Mahalati K, MacDonald AS. Sirolimus-tacrolimus combination immunosuppression. Lancet 2000; 355: 376–377.

15. Mendez R, Gonwa T, Yang HC, Weinstein S, Jensik S, Steinberg S, for the Prograf Study Group. A prospective, randomized trial of tacrolimus in combination with sirolimus or mycophenolate mofetil in kidney transplantation: Results at 1 year. Transplantation 2005; 80: 303–309.

16. Allison AC, Eugui EM. Mycophenolate mofetil and its mechanisms of action. Immunopharmacology 2000; 47: 85–118.

17. Sehgal SN. Rapamune (Sirolimus, rapamycin): An overview and mechanism of action. Ther Drug Monit 1995;17: 660–665.

18. Shihab FS, Bennett WM, Yi H, Choi SO, Andoh TF. Combination therapy with sirolimus and mycophenolate mofetil: Effects on the kidney and on transforming growth factor-beta1. Transplantation 2004; 77: 683–686.

19. Poon M, Marx SO, Gallo R, Badimon JJ, Taubman MB, Marks AR. Rapamycin inhibits vascular smooth muscle cell migration. J Clin Invest 1996; 98: 2277–2283.

20. Dambrin C, Klupp J, Birsan T et al. Sirolimus (rapamycin) monotherapy prevents graft vascular disease in nonhuman primate recipients of orthotopic aortic allografts. Circulation 2003; 107: 2369–2374.

21. Klupp J, Dambrin C, Hibi K et al. Treatment by mycophenolate mofetil of advanced graft vascular disease in non-human primate recipients of orthotopic aortic allografts. Am J Transplant 2003; 3: 817–829.

22. Gregory CR, Huang X, Pratt RE et al. Treatment with rapamycin and mycophenolic acid reduces arterial intimal thickening produced by mechanical injury and allows endothelial replacement. Transplantation 1995; 59: 655–661.

23. Jolicoeur EM, Qi S, Xu D, Dumont L, Daloze P, Chen H. Combination therapy of mycophenolate mofetil and rapamycin in prevention of chronic renal allograft rejection in the rat. Transplantation2003; 75: 54–59.

24. Kreis H, Cisterne JM, Land W *et al*. Sirolimus in association with mycophenolate mofetil induction for the prevention of acute graft rejection in renal allograft recipients. Transplantation 2000; 69: 1252–1260.

25. Flechner SM, Goldfarb D, Modlin C *et al*. Kidney transplantation without calcineurin inhibitor drugs: A prospective, randomized trial of sirolimus versus cyclosporine. Transplantation 2002; 74: 1070–1076.

26. Hamdy AF, El-Agroudy AE, Bakr MA *et al*. Comparison of sirolimus with low-dose tacrolimus versus sirolimus-based calcineurin inhibitor-free regimen in live donor renal transplantation. Am J Transplant 2005; 5: 2531–2538.

27. Flechner SM, Friend PJ, Brockmann J *et al*. Alemtuzumab induction and sirolimus plus mycophenolate mofetil maintenance for CNI and steroid-free kidney transplant immunosuppression. Am J Transplant 2005; 5: 3009–3014.

28. Ekberg H, Tedesco-Silva H, Demirbas A *et al*. Reduced exposure to calcineurin inhibitors in renal transplantation. N Engl J Med 2007; 357: 2562–2575.

29. Lebranchu Y, Thierry A, Toupance O *et al*. Efficacy on renal function of early conversion from cyclosporine to sirolimus 3 months after renal transplantation: CONCEPT study. Am J Transplant 2009; 9: 1115–1123.

30. Schena FP, Pascoe MD, Alberu J *et al*. Conversion from calcineurin inhibitors to sirolimus maintenance therapy in renal allograft recipients: 24-month efficacy and safety results from the CONVERT trial. Transplantation 2009; 87: 233–242.

31. Weir MR, Mulgaonkar S, Chan L, *et al*. Mycophenolate mofetil-based immunosuppression with sirolimus in renal transplantation: a randomized, controlled Spare-the-Nephron trial. *Kidney Int* 2011; 79: 897–907.

32. Larson TS, Dean PG, Stegall MD *et al*. Complete avoidance of calcineurin inhibitors in renal transplantation: A randomized trial comparing sirolimus and tacrolimus. Am J Transplant 2006; 6: 514–522.

33. Augustine JJ, Knauss TC, Schulak JA, Bodziak KA, Siegel C, Hricik DE. Comparative effects of sirolimus and mycophenolate mofetil on erythropoiesis in kidney transplant patients. *Am J Transplant* 2004; 4: 2001–2006.

34. van Gelder T, ter Meulen CG, Hene R, Weimar W, Hoitsma A. Oral ulcers in kidney transplant recipients treated with sirolimus and mycophenolate mofetil. *Transplantation* 2003;75: 788–791.

35. van Gelder T, Shaw L. The rationale for and limitations of therapeutic drug monitoring for mycophenolate mofetil in transplantation. *Transplantation* 2005; 80: S244–S253.

36. Cattaneo D, Merlini S, Zenoni S et al. Influence of co-medication with sirolimus or cyclosporine on mycophenolate acid pharmacokinetics in kidney transplantation. *Am J Transplant* 2005; 5: 2937–2944.
